# One-year clinical evaluation of class II bulk-fill restorations in primary molars: a randomized clinical trial

**DOI:** 10.1590/0103-6440202205069

**Published:** 2022-12-05

**Authors:** Larissa D’Olanda Gindri, Igor Perlin Cassol, Tatiana Tambara Fröhlich, Rachel de Oliveira Rocha

**Affiliations:** 1 Department of Stomatology, Federal University of Santa Maria, Santa Maria, RS, Brazil; 2 Federal University of Santa Maria, Santa Maria, RS, Brazil

**Keywords:** criteria, survival, longevity, class II, primary teeth

## Abstract

This double-blind, randomized clinical trial aimed to compare the clinical performance and clinical time to restore occluso-proximal cavities in primary molars with*bulk-fill*resin and conventional resin. A total of 140 class II restorations in primary molars of 65 participants (mean age of 6.7 + 1.5) were placed in two random groups:*bulk-fill*and conventional resin. The restorations were evaluated using FDI criteria at the baseline, 6-month, and one year by a single calibrated examiner, and the clinical restorative time was measured with a digital timer. The success and survival of the restorations were evaluated with Kaplan-Meier graphs. The log-rank test compared the curves. Differences in restorative clinical time were compared using the Mann-Whitney U test. The level of significance was 5%. After one year, 115 restorations were evaluated. The success probability was 88.7% for Filtek Z350 XT and 85.9% for FiltekTM Bulk-fill, and for the survival probability, Filtek Z350 XT presented 90%, and FiltekTM Bulk-fill presented 93.7%. No significant difference was found between the success and survival curves (p=0.62), (p=0.51). The main reason for failure was marginal adaptation.*Bulk-fill*resin* *required 30% less time than the conventional resin (p<0.001).*Bulk-fill*resin presented similar clinical performance to the conventional resin and required less restorative clinical time. It is an option to restore class II lesions of primary molars.

## Resumo

Este ensaio clínico randomizado, duplo-cego objetivou comparar a performance clínica e tempo clínico para restaurar cavidades ocluso-proximais em molares decíduos, restauradas com resina *bulk-fill* e resina convencional. Um total de 140 restaurações classe II em molares decíduos de 65 participantes (média de idade 6.7 + 1.5) foram realizadas dividas em dois grupos randomizados: resina *bulk-fill* e resina convencional. As restaurações foram avaliadas conforme o critério da FDI no baseline, após 6 meses e 1 ano, por um único examinador calibrado e o tempo clínico restaurador foi mensurado por um cronômetro digital. O sucesso e a sobrevida das restaurações foram avaliados através dos gráficos de Kaplan-Meier. O teste de log-rank comparou as curvas. A diferença no tempo clínico restaurador foi comparada usando o teste U de Mann-Whitney. O nível de significância foi de 5%. Após 1 ano, 115 restaurações foram avaliadas. A probabilidade de sucesso foi de 88,7% para Filtek Z350 XT e 85.9% para Filtek^TM^ Bulk-fill e quanto a probabilidade de sobrevivência, Filtek Z350 XT apresentou 90% e Filtek^TM^ Bulk-fill apresentou 93,7%. Não foi encontrada diferença significativa entre as curvas de sucesso e sobrevida (p=0,62), (p=0,51). A principal causa de falha foi a adaptação marginal. A resina *bulk-fill* exigiu 30% menos tempo do que a resina convencional (p<0,001). A resina *bulk-fill* apresentou desempenho clínico semelhante ao da resina convencional e necessitou menor tempo clínico restaurador. Dessa forma, é uma opção para restaurar lesões classe II em molares decíduos.

## Introduction

The restoration of carious lesions is the most common procedure performed in primary teeth ^1^ ^,^ ^2^. The composite resins have become very popular for direct posterior restorations of primary teeth due to their main advantages as conservative preparations, aesthetic characteristics and their good clinical performance ^2^. However, composite resins are a very sensitive and time-consuming technique ^2^ ^,^ ^3^, so bulk-fill resins are an attractive choice for restoring primary teeth.

*Bulk-fill* resin gained space because of the option to build increments up to 4-5 mm. Therefore, single-increment restorations are possible, decreasing the technical sensitivity and chair time inherent to the incremental technique of conventional resins. The polymerization shrinkage stress is reported as the main limitation of monomeric materials like composite resins ^4^. It is associated with gap formation, cusp deflection, poor marginal adaptation, postoperative sensitivity, and secondary caries ^4^. *Bulk-fill* resins contain stress-relieving monomers and pre-polymerized particles that reduce polymerization shrinkage even when inserted in larger increments than the conventional 2 mm recommended by conventional resin manufacturers ^5^. Likewise, the efficiency of polymerization is also a concern regarding the longevity of restorations, as inefficient polymerization compromises the material's mechanical properties ^6^. For efficient polymerization, *bulk-fill* resins improve translucency, using specific polymerization modulators and more potent initiator systems ^7^.

The results of the clinical performance of bulk-fill composite resins are promising (8-10). In a systematic review and meta-analysis of randomized clinical trials that compared *bulk-fill* resins and conventional resins, the materials showed similar clinical performance, with follow-up for one to ten years ^8^. However, these findings mainly come from studies on permanent teeth, and the results cannot be extrapolated to primary teeth, as there are morphological and chemical differences between them. ^11^. There is a lack of literature about the clinical performance of *bulk-fill* resin in primary teeth; few studies ^12^ ^-^ ^14^ seem to indicate a similar performance of *bulk-fill* resin and other restorative materials such as conventional resin and compomer. However, more randomized clinical trials are needed to indicate the use of *bulk-fill* resin in posterior primary teeth.

Whereas composite resins are the most common direct restorative material used in posterior primary teeth, bulk-fill resins offer technical simplification, which is very useful in pediatric dentistry, especially for non-cooperative children. Therefore, the aim of this double-blind, randomized clinical trial was to compare the clinical behavior of a *bulk-fill* and a conventional resin for a follow-up period of 1 year and compare the clinical time required to restore occlusal-proximal cavities in primary molars. The null hypothesis tested was that there is no difference in the clinical performance of occlusal-proximal restorations in primary teeth placed with these materials.

## Material and methods

### Ethical approval

This study was reviewed and approved by the Ethics Committee of the Federal University of Santa Maria (UFSM; Santa Maria, Brazil) (81118217.7.0000.5346). The participants and their parents or legal guardians were informed about the objectives and procedures of the study and agreed to participate by signing a statement of informed consent. The study was registered in the Brazilian Clinical Trials Registry (REBEC; RBR-329pyp) and is reported following the recommendations of the CONSORT statement ^15^.

### Trial design, settings and location of data collection

A double-blind (patient and evaluator), randomized controlled trial was performed with two parallel groups - the intervention group: *bulk-fill* composite resin, and the control group: conventional composite resin. The study was conducted in the pediatric dentistry clinic of the Federal University of Santa Maria, Rio Grande do Sul, Brazil, from March to November 2018.

### Eligibility criteria

One trained examiner through clinical examination and bitewing radiography recruited eligible patients who sought dental care at the local university. Children aged 5 to 9 years old and presenting at least one primary molar with a moderately deep dentin occlusal-proximal caries lesion, without signs of irreversible pulp pathologies or necrosis, were invited to participate in the study. The lesions should be restricted to the occlusal and proximal surfaces without involving the buccal and lingual walls to standardize the preparations. The presence of the previous restoration in the selected tooth, root resorption over 2/3, children with an adverse medical history, parents who were not available for periodic follow-up, and teeth that it was not possible to use rubber dam isolation were not included in this study.

### Sample size calculation

The success rates of a previous study comparing the survival of restorations placed with the *bulk-fill* flow and conventional composite resin were used for the sample calculation of the present study ^16^. The parameters considered were: α = 5%, power of 80%, considering the outcome binary (success/failure), and equivalence study, with a limit of 10%. The number of restorations was increased to consider the 20% possible drop-out of participants; the minimal sample size was 70 restorations in each group. The sample calculation was performed using a freely available online website (www.sealedenvelope.com).

### Randomization and allocation concealment

The randomization process was carried out on a website (www.sealedenvelope.com) by a researcher (ROR) who did not participate in the operative procedures or the evaluation of the restorations. One hundred and forty cavities were randomized; 70 were assigned to the test group (*bulk-fill* resin) and 70 to the control group (conventional resin) by a randomization list defining the order of the composite resin placement.

The randomization list was generated and transferred into opaque, sealed, and numbered envelopes. The operator only had access to the envelope at the time of insertion of the composite resin into the cavity (after completing the adhesive protocol).

### Blinding

The participants (children) and their caregivers were blind to the restorative material used. The evaluator (TTF) was also blinded and evaluated the restorations without information about which group the restoration was allocated. Blinding of the operator was not possible due to differences in the application of restorative materials. In order to minimize possible biases, the operator only opened the allocation envelope after completing the adhesive protocol, which was the same for all restorations.

### Interventions: restorative treatment

A single operator placed all restorations. The operator was trained by a professor and restored the equivalent of 10% of the sample (14 restorations) before the study started to train the differences between the restorative materials, bulk-fill technique, and incremental technique.

The operator anesthetized the teeth (lidocaine 2% with epinephrine 1:100:000) and performed rubber dam isolation, including at least one tooth adjacent to the occlusal-proximal caries lesion. The cavity design was restricted to access and extension of the caries lesion; the operator did not prepare any additional retention or bevel. The removal of caries tissue followed a selective caries removal up to firm dentine, following coloration and texture parameters ^17^ and complete carious tissue removal from the cavosurface margins and all lateral walls with a slow-speed round bur in a low-speed handpiece and hand instruments. If necessary, access to carious dentine was obtained using a spherical diamond bur mounted in a high-speed handpiece. Before starting the adhesive protocol, cavities dimensions (depth and buccal-lingual distance) were measured with a periodontal millimeter probe. A metallic matrix was placed with wooden wedges, and the cavities were cleaned by thoroughly rinsing with water. All teeth received the same adhesive protocol. The total-etch technique was performed with a 37% phosphoric acid gel (Condac 37, FGM, Joinville, SC, Brazil) for 15 seconds, rinsed for 20 seconds, and gently air-dried. The adhesive system (Scotchbond^TM^ Universal, 3M ESPE, St. Paul, MN, USA) was applied actively to the entire surface for 20 seconds. Next, direct a gentle air stream over the adhesive for 5 seconds and light cured for 10 seconds using a light-curing unit (QHL 75 Curing Light, Dentsply Sirona, Milford, DE, USA) with an intensity of 650 mW/mm^2^. Only at this point, the assistant defined the composite resin to be used by opening the allocation envelope.

In the groups assigned for conventional composite resin (control group), the cavities were filled with Filtek Z350 XT (3M ESPE St. Paul, MN, USA, shade A2D and A2E), using an oblique layering technique in 2-mm increments, each increment was light cured for 20 seconds individually. In the experimental group (*bulk-fill* composite resin), cavities were filled with Filtek^TM^ Bulk Fill (St. Paul, MN, USA, shade A2), in one increment up to 4 mm, light cured for 60 seconds. If the cavity exceeded 4 mm depth, it was filled with two increments, light cured individually for the same time. The occlusion was checked using articulating paper, and the restoration was finished using diamond burs (fine grain diamond burs #3118F; KG Sorensen, São Paulo, SP, Brazil); final polishing was performed with polishing tips (KG Sorensen, Cotia, São Paulo, Brazil). The materials used in this study and the technical sequence are listed in Box 1.

**Box 1:**
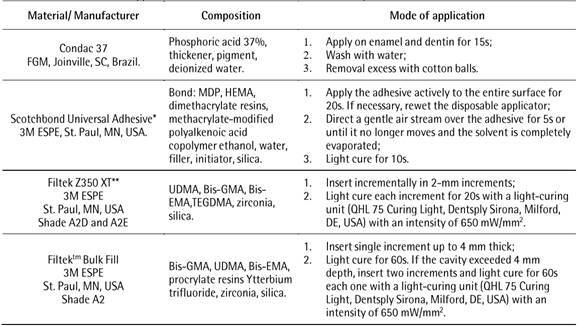
Characteristics and application mode of the materials used in this study

Abbreviations: bis-GMA, bisphenol-A diglycidyl dimethacrylate; UDMA, urethane dimethacrylate; TEGDMA, triethylene glycol dimethacrylate; bis-EMA, ethoxylated bisphenol A dimethacrylate. * Also known as Single Bond Universal in some countries. ** Also known as Filtek Supreme Plus in some countries.

## Clinical evaluation

A single examiner performed the evaluation of the restorations using the World Dental Federation (FDI) criteria ^18^. The examiner was trained about the FDI criteria. In the first moment, the evaluated criteria were presented with photographic images of restorations and discussed. Afterward, the examiner evaluated a random sequence of restoration images two times with an interval of 7 days, and the assigned scores were compared to those assigned by a reference examiner ('gold standard'). Cohen's kappa test demonstrated a kappa value <0.85 intra-examiner.

The restorations were evaluated at the baseline (until one month), six-month, and one-year after being placed. The following items were evaluated: 1) functional properties - fracture of restorative material/restoration retention, and marginal adaptation; 2) aesthetic properties - surface gloss, surface staining, marginal staining, and anatomical form; 3) biological properties - recurrence of caries. These items were ranked according to the scores: 1) clinically very good; 2) clinically good; 3) clinically satisfactory; 4) clinically unsatisfactory (but can be repaired), and 5) clinically poor (should be replaced) ^18^. In each time evaluated, visible plaque index and gingival bleeding index evaluations were performed, and professional plaque removal. In addition, guidance about oral hygiene and dietary habits was reinforced for parents and patients.

### Statistical analysis

The primary outcome was the restoration failure according to the resin composite after a one-year follow-up. The restorations with FDI scores 4 and 5 (clinically unsatisfactory and clinically poor, respectively) and teeth that required endodontic treatment were considered as failure in the success analysis. Exfoliated teeth during the follow-up period without symptoms were considered a clinical success. In the survival analysis, only restorations classified in score 5 were considered as failures ^19^. The success and survival of the restorations concerning composite resin were evaluated with survival tables and Kaplan-Meier graphs. The log-rank test compared the success and survival curves.

Cavities dimensions (depth and buccal-lingual distance) and clinical restorative time were calculated and are presented as the mean and standard deviation. The differences in restorative clinical time and cavities dimensions (depth and buccal-lingual distance) between the *bulk-fill* and conventional resin composite groups were compared using the Mann-Whitney U test, as the data was not normality distributed (Anderson-Darling test).

The data were analyzed using Minitab software, version 19 (Minitab Inc., State College, PA, USA), with a significance level of 5%.

## Results

**Figure 1:**
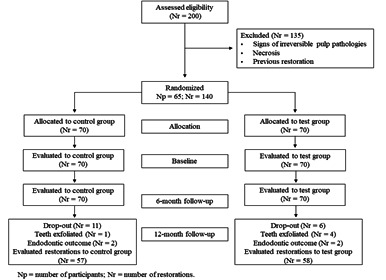
CONSORT flowchart of the participants’ progress through the trial phases.

A total of 140 restorations were placed in 65 children (39 boys **-** 26 girls) with a mean age of 6.7 + 1.5 (SD), presenting a decayed, missing, and failed primary teeth (dmft) index mean of 5.8 + 2.4. Table 1 shows the socioeconomic, demographic, and clinical variables of patients according to experimental group. At the baseline and 6-month follow-up, 140 restorations were evaluated according to the summarized FDI criteria. After one year, 5 teeth were lost due to physiological exfoliation, 7 patients who received 16 restorations did not return (drop-out 11.4%), and 4 teeth needed endodontic treatment. Finally, 115 restorations were evaluated after follow-up (Figure 1).

**Figure 2:**
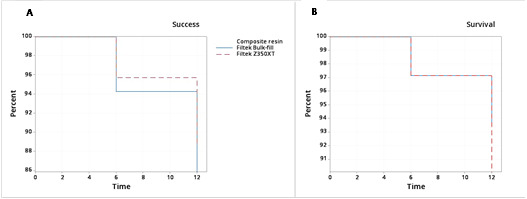
Success (A) and survival (B) curves (Kaplan-Meier) for Bulk-fill and conventional resin restorations over one-year, log-rank p = 0,62 (A) and p = 0,51 (B).

The success probability after 12 months was 88.7% for Filtek Z350 XT and 85.9% for Filtek^TM^ Bulk-fill. The mean success time for Filtek Z350 XT restorations was 11.7 and for Filtek Bulk-fill was 11.6 months. There was no significant difference in the success curves after 12 months (p=0.62) (Figure 2). Considering the survival probability, Filtek Z350 XT presented 90%, and Filtek Bulk-fill presented 93.7% after 12 months. The mean survival time was 11.8 for both composites. No significant difference was found between the survival curves (p=0.51) (Figure 2).

Table 2 summarized the distribution of the restorations according to the evaluated parameters of the FDI criteria. Twelve restorations, 7 of *bulk-fill* resin, and 5 of conventional resin, received scores 4 or 5 at the end of one-year follow-up and needed intervention. Marginal adaptation was the main cause of failure (5 of Filtek Bulk-fill and 2 of Filtek Z350 XT), followed by fracture/retention (1 of Filtek Bulk-fill resin and 2 of Filtek Z350 XT) and the recurrence of caries (1 restoration in each group). Among the 5 failure restorations placed with conventional resin, only the restoration that failed due to marginal adaptation can be repaired (score 4); the other 4 restorations required replacement (score 5). Among the 7 failed restorations placed with *bulk-fill* resin, 5 received repairs (4 failed due to marginal adaptation and 1 failed due to recurrence of pathology), and 2 needed to be replaced. No restoration required intervention due to aesthetic parameters; only minor changes were observed during the 12-month follow-up. A total of 4 teeth required endodontic treatment - 2 restored with conventional resin and 2 with *bulk-fill* resin.

The results for clinical restorative time, number of increments, and cavity dimensions for *bulk-fill* and conventional resin composites are presented in table 3. The clinical restorative time using a bulk-fill resin was almost 30% shorter, corresponding to approximately 2 minutes faster (p<0.001) as fewer increments were used (p<0.001). No statistically significant differences were found between the composites concerning the cavity dimensions (p=0.113, and p=0.255 to depth and buccal-lingual distance, respectively).

**Table 1:** Baseline characteristics of the subjects included into the study groups

Characteristics of sample	Conventional resin (Filtek Z350 XT) (Nr = 70)	***Bulk-fill* resin (Filtek** ^TM^ **Bulk Fill) (Nr = 70)**
Age (years) - Mean (SD)	6,9 + 1,5	6,5 + 1,5
*Dmft* ^*^ - Mean (SD)	5,9 + 2,6	5,6 + 2,2
Sex - n (%)		
Female	24 (34)	27 (39)
Male	46 (66)	43 (61)
Teeth - n (%)		
First molar	39 (56)	36 (51)
Second molar	31 (44)	34 (49)
Arch - n (%)		
Upper	36 (51)	30 (43)
Lower	34 (49)	40 (57)
Mother education level - n (%)		
Primary school	30 (43)	22 (31)
High school	38 (54)	42 (60)
Graduate	2 (3)	6 (9)
Family income - n (%)^**^		
≤ 2 BMW	41 (59)	41 (59)
> 2 BMW	29 (41)	29 (41)
Skin color - n (%)		
White	42 (60)	51 (73)
Not white	28 (40)	19 (27)
VPI^***^ - n (%)		
< 10%	22 (31)	20 (29)
≤10% - ≥ 30%	44 (63)	47 (67)
> 30%	4 (6)	3 (4)
GBI^***^ - n (%)		
< 10%	31 (44)	26 (37)
≤10% - ≥ 30%	38 (54)	42 (60)
> 30%	1 (1)	2 (3)

Nr = number of restorations; *dmft = decayed, missing, and filled deciduous teeth index; ** measured through the Brazilian Minimum Wage (BMW) (1 BMW corresponded to approximately USD 250 during the period of data collection), all families of children included in the study received less than 3 BMW. ***Index collected in the last evaluation.

**Table 2:** Evaluation of the restorations according to FDI criteria used in this study

	Conventional resin (Filtek Z350 XT)			***Bulk-fill* resin (Filtek** ^TM^ **Bulk Fill)**		
	Baseline	6-month	1 year	Baseline	6-month	1 year
Surface gloss (1/2/3/4/5)	40/30/0/0/0	33/34/0/0/0	17/33/1/0/0	46/24/0/0/0	37/29/0/0/0	25/26/0/0/0
Surface staining (1/2/3/4/5)	70/0/0/0/0	67/0/0/0/0	51/0/0/0/0	69/1/0/0/0	65/1/0/0/0	49/2/0/0/0
Marginal staining (1/2/3/4/5)	70/0/0/0/0	65/2/0/0/0	49/2/0/0/0	68/2/0/0/0	63/3/0/0/0	48/3/0/0/0
Anatomical form (1/2/3/4/5)	49/21/0/0/0	46/21/0/0/0	27/21/3/0/0	58/12/0/0/0	50/16/0/0/0	33/17/1/0/0
Fracture/ retention (1/2/3/4/5)	70/0/0/0/0	67/0/0/0/1	51/0/0/0/2	70/0/0/0/0	65/1/0/0/1	51/0/0/0/1
Marginal adaptation (1/2/3/4/5)	70/0/0/0/0	67/0/0/1/1	49/2/0/1/1	69/1/0/0/0	62/4/0/2/1	49/2/0/4/1
Recurrence of caries (1/2/3/4/5)	70/0/0/0/0	67/0/0/0/0	51/0/0/0/1	70/0/0/0/0	66/0/0/0/0	51/0/0/1/0

The numbers separated by slash represent the number of evaluated restorations that received the respective score: 1. clinically very good; 2. clinically good; 3. clinically satisfactory; 4. clinically unsatisfactory; 5. clinically poor

**Table 3:** Means and standard deviations considering restorative time, number of increments and other variables for filling occluso-proximal cavities in primary molars

	Conventional resin (Filtek Z350 XT)	***Bulk-fill* resin** **(Filtek** ^TM^ **Bulk Fill)**	Significance Mann-Whitney U-test
Clinical restorative time^*^	6.1 (3.2)	4.4 (2.2)	p<0.0001
Depth^**^	2.7 (0,9)	2.4 (0.9)	p=0.1133
Buccal-lingual distance^**^	2.7 (0.9)	2.9 (0.9)	p=0.255

* minutes ** mm

## Discussion

In the present study, the clinical performance of two composite resins in class II restorations placed on primary molars was compared, and similar clinical performance between *bulk-fill* and conventional composite resin was demonstrated; thus, the null hypothesis that there is no difference between the restorations was accepted. Furthermore, this study also evaluated the clinical time required to place conventional resin restorations using the incremental technique and bulk-fill resin in a single increment. Restorations placed with *bulk-fill* resin were significantly faster than restorations placed with conventional resin.

Several clinical studies available on the clinical performance of *bulk-fill* resin, including meta-analyses, have shown its good performance ^8^ ^-^ ^10^ ^,^ ^16^ ^,^ ^20^; however, in primary teeth, there are still few randomized clinical trials ^12^ ^-^ ^14^, especially those that compare conventional resin and *bulk-fill* resin ^12^ ^,^ ^14^. The main advantage attributed to *bulk-fill* resin concerns clinical time, in which its reduction is significant in pediatric dentistry. When the material of choice is composite resin, patient-related factors, especially in the case of non-cooperative children, can affect the performance of the restoration due to the sensitive technique ^2^. The reduction of clinical time can minimize these adversities. This study is the first to evaluate both clinical performance and clinical time of the use of *bulk-fill* resin in primary teeth, and the compilation of results - similar clinical performance and shorter clinical time of *bulk-fill* restorations - showed an advantage the use of *bulk-fill* resin in the care of children compared to conventional resins.

Only a few recent randomized clinical trials evaluated *bulk-fill* resin in primary teeth ^9^ ^-^ ^11^. Ehlers et al. ^13^ compared class II restorations placed with *flowable bulk-fill* resin *vs.* a compomer. Öter et al. ^12^ used class I restorations to compare *bulk-fill* resin and conventional composite resin, and Akman et al. ^14^ evaluated a glass ionomer restorative system, two *bulk-fill* resins, and a conventional resin in class II restorations. All studies follow-up the restorations for one year and the *bulk-fill* resin showed similar clinical behavior to other restorative materials in all studies, except for glass ionomer cement, which was statistically less successful in marginal adaptation and retention criteria ^14^. The difference in restorative materials (compomer, *flowable bulk-fill*, regular *bulk-fill*, conventional resin, and glass ionomer cement), and the type of lesion (class I or class II) used in the studies do not allow the results to be compared. Our results are comparable to those of Akman et al. ^14^, which also compared the*bulk-fill*resin and the conventional resin in occlusal-proximal cavities in primary molars. Both studies showed similar clinical performance between *bulk-fill* resin and conventional resin. The most encouraging result found in RCTs that evaluated *bulk-fill* in primary molars is that the clinical performance of *bulk-fill* resin is comparable to conventional resin and compomer.

After a one-year clinical follow-up, 115 restorations were evaluated according to the FDI criteria, and 12 restorations required some reintervention. The success rates of restorations were 88.7% for conventional resin and 85.9% for *bulk-fill* resin. Despite the number of failures in a short follow-up period, the materials tested in the study were two composite resins, so the results were expected to be similar to the composite resin in other clinical studies that evaluated the clinical behavior of direct posterior restorations in primary teeth ^21^ ^,^ ^22^. Sengul and Gurbuz ^18^ evaluated class II restorations placed with composite resin, compomer, GIC, and RMGIC. The estimative success rate of composite resin restorations was 85% and 79.5% at one-year and 2-year follow-up, similar to our findings. Bektas et al. ^22^ also found similar estimative success rates for conventional resin in class II restorations, 85% and 80.6% at one-year and 18-month follow-up, respectively.

Only class II cavities were included in the sample. Occlusal-proximal restorations have a trend for more failure than restorations restricted to the occlusal surface ^2^ ^,^ ^23^. The challenges in class II cavities are related to the size of the cavity and the depth, especially in primary molars. Due to the shape of the proximal surfaces of the primary molars, large proximal cavities result in preparations with limited mechanical retention ^24^. Even so, the dimensions of the cavities were similar in depth and buccal-lingual distance between the cavities restored with conventional and *bulk-fill* resin; thus, the technical difficulty was the same regardless of the restorative material.

To the best of our knowledge, this is the first randomized clinical trial that tested *bulk-fill* resin in primary molar under selective caries removal. An *in vitro* study evaluated the cusp deflection, presence of enamel cracks, and fracture resistance of class II restorations placed with *bulk-fill* resin in permanent molars. The results showed that the selective caries removal did not influence the biomechanical behavior of restorations ^25^. Selective caries removal has been strongly recommended as being less invasive, reducing the risks of pulp exposure and postoperative sensitivity ^17^. Nevertheless, it may increase the risk of restoration failure ^26^. In addition, in class II cavities, the technique becomes more complex, so achieving adequate sealing in the proximal margins of the restoration is challenging ^24^ ^,^ ^26^. Still, minimally invasive approaches like selective caries removal should be preferable to total caries removal, and patients should be recalled at shorter intervals so that restorations can be repaired before needing replacement ^26^.

The decision to use the FDI criteria was based on the possibility of considering a restoration classified as clinically unacceptable (score 4) to be repairable ^18^. Therefore, the success analysis (scores 1, 2, 3) and survival analysis (scores 1, 2, 3, 4) were performed ^19^. In the survival analysis, restorations that only needed repair were considered acceptable ^18^; thus, the survival rate of the restorations was higher than the success rate, as half of the restorations that failed needed only repairs. It is essential to highlight that the bulk-fill restorations success rate was 85.9% and increased to 93.7% in the survival analysis, with 7 failures of the *bulk-fill* resin, five considered as repairable, and only 2 needing replacement. Although there is no significant difference between the materials, this result may still be clinically relevant. Restoration repair increases the survival of restorations, which is especially important for primary teeth, as repair is less invasive, comfortable, and inexpensive than restoration replacement ^18^ ^,^ ^26^.

Stress due to polymerization shrinkage and inadequate polymerization may lead to the debonding of material from the cavity walls and subsequent micro-gaps formation (4). A recent systematic review and meta-analysis ^(^ ^8^ compared *bulk-fill* and conventional resins in terms of their physical-mechanical properties, and clinical performance pointed out that *bulk-fill* resins have less polymerization shrinkage stress, cusp deflection, and microhardness than conventional composites, and both materials presented similar marginal quality, flexural strength, and fracture strength. Despite these *in vitro* results, the clinical performance of *bulk-fill* and conventional resins was similar in randomized clinical trials. However, a meta-analysis in permanent teeth performed by Kruly et al. ^20^ found better results for marginal adaptation after 12 months in conventional resin restorations than low polymerization shrinkage composite resin, including bulk-fill resins. In our study, marginal adaptation and fracture/retention was the main reasons for failures, for both groups.

This study included 65 children who received 140 restorations. These patients belong to a low socioeconomic level, having a high caries risk. Factors related to the patient, such as socioeconomic status, caries risk, and oral hygiene, were shown to influence the failure of restorations ^2^ ^,^ ^24^. A retrospective study assessed the potential factors associated with treatment failure of selective caries removal in the primary teeth of children with high caries experience. It demonstrated the association between high dmft and biofilm accumulations and a higher risk of treatment failure ^27^. Despite the characteristics of the sample, recurrence of caries was the reason for failure in only two restorations, probably due to all children being included in a program of periodic recalls, reinforcing oral hygiene care and dietary habits. On the other hand, 4 restorations required endodontic intervention. These patients already had previous endodontic treatments or extractions due to caries disease; the endodontic outcome is probably not related to the restorative material but factors related to the patient.

As previously described, the cavities presented similar buccal-lingual dimensions and depth. Thus, the technical difficulty was the same in the groups. Even so, the bulk-fill resin showed a shorter restorative clinical time. This result shows that filling the cavity in a single increment is easier to handle and more straightforward than the conventional incremental technique.

In this study, factors influencing restorations' longevity were minimized. All restorations were performed under local anesthesia and a rubber dam, minimizing the influence of the child's behavior. ^2^. All cavities received the universal adhesive system Scotchbond Universal^TM^ in etch-and-rinse mode, which is recommended to restore primary molars after selective caries removal ^28^. Even so, shorter restorative clinical time in the bulk-fill group should be associated with technical simplification and easy handling.

The limitations of this study are the short follow-up period (one year); however, in primary teeth, physiological exfoliation hinders more extended periods of follow-up, so the period of 1-year follow-up becomes relevant. In addition, due to the inability to introduce a blind operator, to minimize bias, the cavity was only allocated to one of the experimental groups, after completing the adhesive protocol. Drop-outs are usually common complications in longitudinal studies, especially those involving children. Finally, this study was conducted at a university; all restorations were placed in ideal conditions, resulting in high internal validity; however, it may not reflect clinical practice.

*Bulk-fill* resin and conventional resin showed similar clinical performance after one year. Furthermore, both composite resins showed good clinical behavior. The significant reduction of restorative clinical time when a *bulk-fill* resin was used represents an advantage over conventional resins, especially in pediatric and non-cooperative patients. Therefore, the bulk-fill resin is recommended to restore occlusal-proximal lesions in primary molars.

## References

[1] Ricketts D, Lamont T, Innes NP, Kidd E, Clarkson JE (2013). Operative caries management in adults and children. Cochrane Database Syst Rev.

[2] Chisini LA, Collares K, Cademartori MG, Oliveira LJCde, Conde MCM, Demarco FF (2018). Restorations in primary teeth: a systematic review on survival and reasons for failures. Inter J Paediatr Dent.

[3] Ferracane JL (2008). Buonocore Lecture. Placing dental composites--a stressful experience. Oper Dent.

[4] Campos EA, Ardu S, Lefever D, Jassé FF, Bortolotto T, Krejci I (2014). Marginal adaptation of class II cavities restored with bulk-fill composites. J Dent.

[5] Fronza BM, Rueggeberg FA, Braga RR, Mogilevych B, Soares LE, Martin AA (2015). Monomer conversion, microhardness, internal marginal adaptation, and shrinkage stress of bulk-fill resin composites. Dent Mater.

[6] Durner J, Obermaier J, Draenert M, Ilie N (2012). Correlation of the degree of conversion with the amount of elutable substances in nanohybrid dental composites. Dent Mater.

[7] Bucuta S, Ilie N (2014). Light transmittance and micro-mechanical properties of bulk fill vs. conventional resin based composites. Clin Oral Investig.

[8] Cidreira Boaro LC, Pereira Lopes D, de Souza ASC, Lie Nakano E, Ayala Perez MD, Pfeifer CS, Gonçalves F (2019). Clinical performance and chemical-physical properties of bulk fill composites resin - a systematic review and meta-analysis. Dent Mater.

[9] Veloso SRM, Lemos CAA, de Moraes SLD, do Egito Vasconcelos BC, Pellizzer EP, de Melo Monteiro GQ (2019). Clinical performance of bulk-fill and conventional resin composite restorations in posterior teeth: a systematic review and meta-analysis. Clin Oral Investig.

[10] Arbildo-Vega HI, Lapinska B, Panda S, Lamas-Lara C, Khan AS, Lukomska-Szymanska M (2020). Clinical Effectiveness of Bulk-Fill and Conventional Resin Composite Restorations: Systematic Review and Meta-Analysis. Polymers (Basel)..

[11] Oliveira MAHM, Torres CP, Silva JMG, Chinelatti MA, De Menezes FC, Palma-Dibb RG (2010). Microstructure and Mineral Composition of Dental Enamel of Permanent and Primary Teeth. Microsc Res Tech.

[12] Öter B, Deniz K, Çehreli SB (2018). Preliminary data on clinical performance of bulk-fill restorations in primary molars. Niger J Clin Pract.

[13] Ehlers V, Gran K, Callaway A, Azrak B, Ernst CP (2019). One-year Clinical Performance of Flowable Bulk-fill Composite vs Conventional Compomer Restorations in Primary Molars. J Adhes Dent.

[14] Akman H, Tosun G (2020). Clinical evaluation of bulk-fill resins and glass ionomer restorative materials: A 1-year follow-up randomized clinical trial in children. Niger J Clin Pract.

[15] Schulz FK, Altman DG, Moher D (2010). CONSORT 2010 Statement: updated guidelines for reporting parallel group randomised trials. BMJ.

[16] Van Dijken JWV, Pallesen U (2017). Bulk-filled posterior resin restorations based on stress-decreasing resin technology: a randomized, controlled 6-year evaluation. Eur J Oral Sci.

[17] Schwendicke F, Frencken JE, Bjorndal L, Maltz M, Manton DJ, Ricketts D (2016). Carious Lesions: Consensus Recommendations on Carious Tissue Removal. Adv Dent Res.

[18] Hickel R, Roulet JF, Bayne S, Heintze SD, Mjör IA, Peters M (2007). Recommendations for conducting controlled clinical studies of dental restorative materials. Clin Oral Investig.

[19] Laske M, Opdam NJM, Bronkhorst EM, Braspenning JCC, Huysmans MCDNJM (2019). The differences between three performance measures on dental restorations, clinical success, survival and failure: A matter of perspective. Dent Mater.

[20] Kruly PC, Giannini M, Pascotto RC, Tokubo LM, Suga U, Marques A (2018). Meta-analysis of the clinical behavior of posterior direct resin restorations: Low polymerization shrinkage resin in comparison to methacrylate composite resin. PLoS One.

[21] Sengul F, Gurbuz T (2015). Clinical Evaluation of Restorative Materials in Primary Teeth Class II Lesions. J Clin Pediatr Dent.

[22] Bektas SD, Uysal S, Dolgun A, Turgut MD (2016). Clinical performance of aesthetic restorative materials in primary teeth according to the FDI criteria. Eur J Paediatr Dent.

[23] Opdam NJ, Van de Sande FH, Bronkhorst E, Cenci MS, Bottenberg P, Pallesen U (2014). Longevity of posterior composite restorations: a systematic review and meta-analysis. J Dent Res.

[24] Franzon R, Opdam NJ, Guimarães LF, Demarco FF, Casagrande L, Haas AN, Araujo FB (2015). Randomized controlled clinical trial of the 24-months survival of composite resin restorations after one-step incomplete and complete excavation on primary teeth. J Dent.

[25] Silva PFD, Oliveira LRS, Braga SSL, Signori C, Armstrong SR, Soares CJ (2018). Effect of selective carious tissue removal on biomechanical behavior of class II bulk-fill dental composite restorations. Dent Mater.

[26] Pedrotti D, Cavalheiro CP, Casagrande L, de Araújo FB, Imparato JCP, Rocha RO (2019). Does selective carious tissue removal of soft dentin increase the restorative failure risk in primary teeth?: Systematic review and meta-analysis. J Am Dent Assoc.

[27] Melgar XC, Opdam NJM, Britto Correa M, Franzon R, Demarco FF, Araujo FB, Casagrande L (2017). Survival and Associated Risk Factors of Selective Caries Removal Treatments in Primary Teeth: A Retrospective Study in a High Caries Risk Population. Caries Res.

[28] Lenzi TL, Pires CW, Soares FZM, Raggio DP, Ardenghi TM, de Oliveira Rocha R (2017). Performance of Universal Adhesive in Primary Molars After Selective Removal of Carious Tissue: An 18-Month Randomized Clinical Trial. Pediatr Dent.

